# Culture-Associated DNA Methylation Changes Impact on Cellular Function of Human Intestinal Organoids

**DOI:** 10.1016/j.jcmgh.2022.08.008

**Published:** 2022-08-28

**Authors:** Rachel D. Edgar, Francesca Perrone, April R. Foster, Felicity Payne, Sophia Lewis, Komal M. Nayak, Judith Kraiczy, Aurélie Cenier, Franco Torrente, Camilla Salvestrini, Robert Heuschkel, Kai O. Hensel, Rebecca Harris, D. Leanne Jones, Daniel R. Zerbino, Matthias Zilbauer

**Affiliations:** 1 European Molecular Biology Laboratory, European Bioinformatics Institute, Wellcome Genome Campus, Hinxton, Cambridge, United Kingdom; 2 Department of Paediatrics, University of Cambridge, Addenbrooke’s Hospital, Cambridge, United Kingdom; 3 Centre for Pathway Analysis, Milner Therapeutics Institute, University of Cambridge, Cambridge, United Kingdom; 4 Department of Paediatric Gastroenterology, Hepatology and Nutrition, Cambridge University Hospitals, Addenbrooke’s Hospital, Cambridge, United Kingdom; 5 Department of Molecular, Cell and Developmental Biology, University of California Los Angeles, Los Angeles, California; 6 Eli and Edythe Broad Stem Cell Research Center, University of California Los Angeles, Los Angeles, California; 7 Witten/Herdecke University, Department of Paediatrics, Helios Medical Centre Wuppertal, Children's Hospital, Wuppertal, Germany; 8 Department of Anatomy and Medicine, Division of Geriatrics, University of California, San Francisco, San Francisco, California; 9 Eli and Edythe Broad Center for Regeneration Medicine, University of California, San Francisco, San Francisco, California; 10 Wellcome Trust–Medical Research Council Stem Cell Institute, University of Cambridge, Cambridge, United Kingdom

**Keywords:** Organoid, Epigenetics, Culture Conditions, Intestinal Epithelium, cDMR, differentially methylated regions in colon cancer, CpG, cytosine-phosphate-guanine dinucleotides, CTCF, CCCTC-binding factor, DNAm, DNA methylation, FDR, false discovery rate, GO, Gene Ontology, IEO, intestinal epithelial organoid, IFNγ, interferon γ, PC, Principal componen, PCA, principal component analysis, TF, transcription factor, TI, terminal ileum, TNFα, tumor necrosis factor α

## Abstract

**Background & Aims:**

Human intestinal epithelial organoids (IEOs) are a powerful tool to model major aspects of intestinal development, health, and diseases because patient-derived cultures retain many features found in vivo. A necessary aspect of the organoid model is the requirement to expand cultures in vitro through several rounds of passaging. This is of concern because the passaging of cells has been shown to affect cell morphology, ploidy, and function.

**Methods:**

Here, we analyzed 173 human IEO lines derived from the small and large bowel and examined the effect of culture duration on DNA methylation (DNAm). Furthermore, we tested the potential impact of DNAm changes on gene expression and cellular function.

**Results:**

Our analyses show a reproducible effect of culture duration on DNAm in a large discovery cohort as well as 2 publicly available validation cohorts generated in different laboratories. Although methylation changes were seen in only approximately 8% of tested cytosine-phosphate-guanine dinucleotides (CpGs) and global cellular function remained stable, a subset of methylation changes correlated with altered gene expression at baseline as well as in response to inflammatory cytokine exposure and withdrawal of Wnt agonists. Importantly, epigenetic changes were found to be enriched in genomic regions associated with colonic cancer and distant to the site of replication, indicating similarities to malignant transformation.

**Conclusions:**

Our study shows distinct culture-associated epigenetic changes in mucosa-derived human IEOs, some of which appear to impact gene transcriptomic and cellular function. These findings highlight the need for future studies in this area and the importance of considering passage number as a potentially confounding factor.

## Graphical abstract

**Figure:**
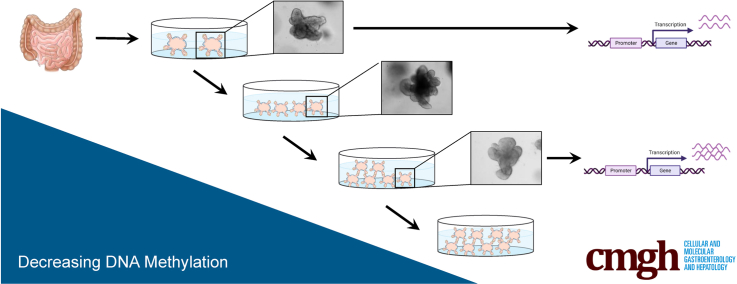

**Summary**

This work describes cell culture–induced changes to DNA methylation, gene expression, and cellular function in human intestinal epithelial organoids. Globally, organoids lost DNA methylation with time in culture while DNA methylation also became generally more variable. This work suggests a shifted epigenetic profile in organoids cultured long term.

Organoids are self-organizing, 3-dimensional structures that are derived from either pluripotent or somatic, organ-specific stem cells. They have been shown to closely mimic both anatomy and cellular function of the in vivo organ. Importantly, the ability to generate such organoids from human cells has turned them into powerful translational research tools with a wide range of applications, including the development of new therapeutics, testing of existing drugs, as well as the application of a personalized treatment approach in several diseases.^1^, ^2^, ^3^, ^4^, ^5^

Among the most advanced human organoid models are mucosa-derived intestinal epithelial organoids (IEOs).^6^ Isolation of Leucine-rich repeat-containing G-protein coupled receptor 5 (LGR5+) intestinal stem cells or entire crypts followed by their culture in an environment closely mimicking the in vivo stem cell niche leads to the development of 3-dimensional mini-organs containing all epithelial cell subsets organized in a crypt-villus structure, closely reflecting the in vivo situation. Mucosal IEOs have been generated successfully from all parts of the digestive tract and from a wide range of donors including healthy individuals of different age groups and patients with intestinal diseases such as inflammatory bowel disease.^7^, ^8^, ^9^, ^10^, ^11^ The latter provides unprecedented opportunities to investigate disease pathogenesis and develop novel treatment approaches using patient-derived organoids.

Another major advantage of human IEOs is the ability to keep them in culture over prolonged time periods (ie, months or even years). Indeed, prolonged culture periods also are required to sufficiently expand organoids before their experimental use. As a result, cellular cultures must undergo numerous rounds of passaging, a process during which organoids are dissociated into individual crypts that give rise to new organoids, thereby increasing their total number. The potential impact of prolonged in vitro culturing of a human mucosa–derived IEO remain largely unknown. Although previous studies have shown a high degree of genetic stability over time,^12^ little is known about epigenetic alterations or changes in cellular function. Despite major progress in optimizing culturing methods aimed at closely mimicking the in vivo situation, fundamental differences such as the absence of gut microbiota or signaling from other cell types (eg, immune or mesenchymal cells) may cause alterations in both cellular epigenome and/or function, as has been shown in other cell culture models.^13^^,^^14^ Understanding the potential impact of prolonged culturing and repeated passaging on cellular function of human IEOs therefore is of critical importance because any changes may confound experimental results as well as impact on their potential use in the field of regenerative medicine.

DNA methylation (DNAm) is one of the main epigenetic mechanisms known to play a key role in regulating cellular function of human cells including the intestinal epithelium.^7^^,^^8^^,^^15^, ^16^, ^17^, ^18^ We previously reported that gut segment–specific DNAm signatures are faithfully retained in human mucosa–derived IEOs and that they are critical for region-specific cellular function.^10^^,^^19^ Although such gut segment–specific DNAm signatures were found to be highly stable over time in IEOs derived from children and adults, human fetal gut–derived IEOs showed substantial changes in their DNAm profiles during prolonged culturing, suggesting a degree of in vitro maturation.

Importantly, epigenetic instability in intestinal cells also has been seen in colon carcinogenesis, with substantial genome-wide DNAm changes observed in colorectal cancer.^20^ Specifically, when compared with healthy colonic mucosa, colorectal cancer shows both losses and gains of DNAm, as well as increased variability in DNAm.^20^, ^21^, ^22^

Here, we set out to monitor global DNAm in human IEOs during prolonged in vitro culture and investigate the impact of associated epigenetic changes on cellular function. Based on the analyses of 173 human mucosa–derived IEOs, we have identified distinct culture-associated DNAm changes, some of which impact gene transcription and cellular function. This highlights the importance of considering culture duration in experimental design and interpretation of results.

## Results

### Prolonged In Vitro Culture of Human IEOs Is Associated With Distinct DNAm Changes

**Figure 1:**
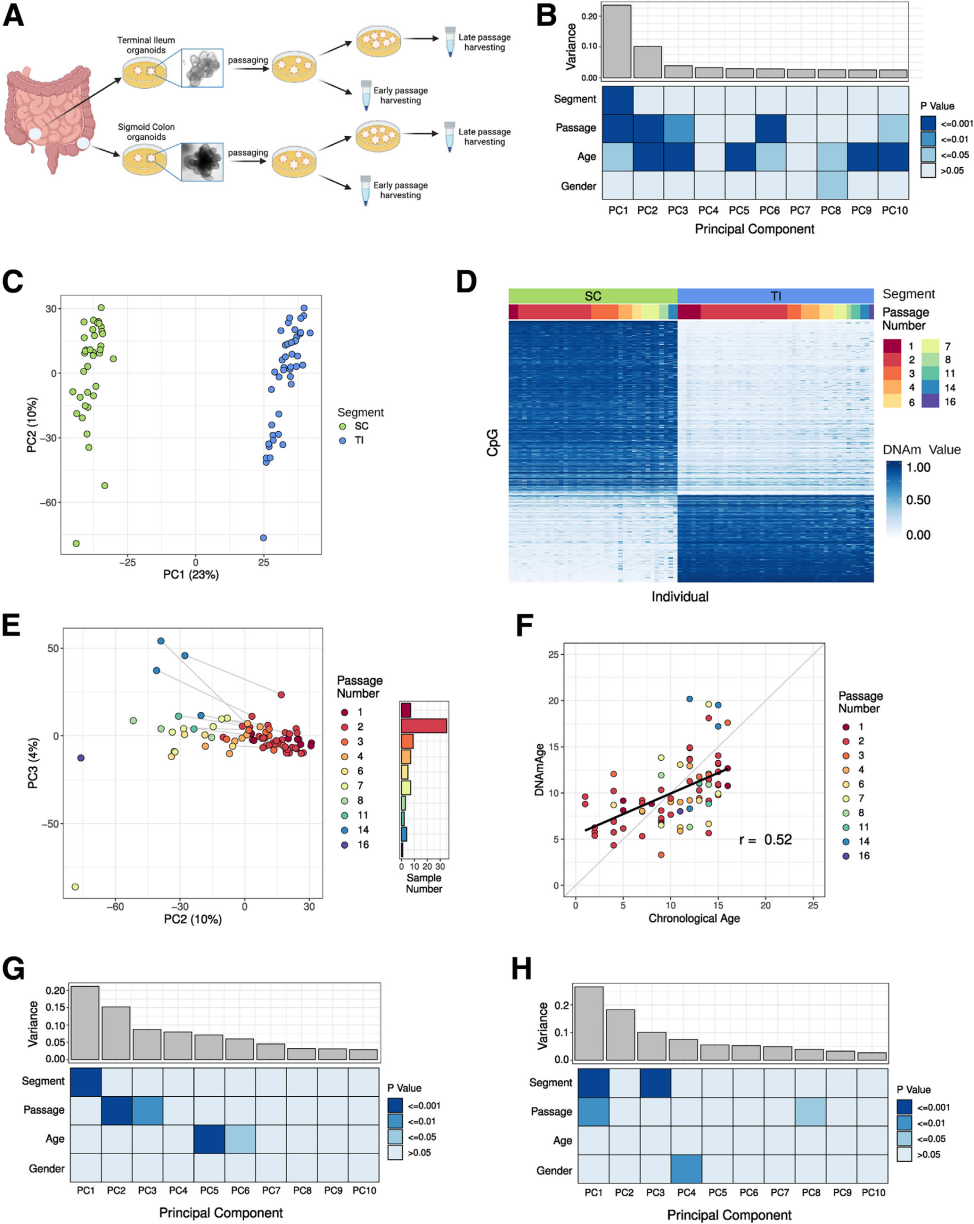
**Sampling site of origin and IEO passage number are associated with the main components of DNAm variation.** (*A*) Outline of study design. (*B*) Scree plot showing DNAm variance accounted for by each PC and association with sample variables. *P* values were generated with a Spearman correlation for continuous variables or an analysis of variance for categoric variables. (*C*) PC1 and PC2 of IEO genome-wide DNAm profiles (cohort 1). (*D*) Heatmap showing DNAm of the top 500 CpGs differentially DNAm between small- and large-bowel IEOs. (*E*) PC2 and PC3 of IEO DNAm profiles. Samples are colored by passage number. *Lines* connect samples derived from the same patient profiled at different passages. (*F*) The association between chronological and epigenetic age is shown, with points colored by the passage of the IEO. As in panel *B*, variance was accounted for by PCs in validation cohorts. (*G*) Cohort 2. (*H*) Cohort 3. SC, sigmoid colon.

To examine the potential impact of prolonged in vitro culture on human IEO DNAm, we recruited a total of 46 children undergoing routine endoscopy and obtained mucosal biopsy specimens from the distal small bowel (ie, terminal ileum [TI]) and distal large bowel (sigmoid colon). Human IEOs were generated (N = 80, cohort 1) (Table 1) and cultured over several months while documenting the number of passages as an indication for culture duration (Figure 1*A*). IEOs were harvested at various time points ranging from passage 1 (approximately 7–10 days) to passage 16 (approximately 4–10 months of culture duration), and genome-wide DNAm profiling was performed. First, we assessed DNAm profiles for any variation and potential association with specific phenotypes or donor characteristics. We therefore performed variance decomposition analyses. Briefly, principal component analysis (PCA) was performed to uncover variance of IEO DNAm profiles followed by testing for a potential correlation between observed DNAm differences and phenotypes (see the Methods section). As shown in Figure 1*B*, IEO DNAm was strongly associated with gut segment (Figure 1*C*) and age. As reported previously by our group,^8^ IEOs faithfully retain gut segment–specific DNAm (ie, small bowel vs large bowel), with differences being stable over prolonged culture duration (Figure 1*C* and *D*). A significant correlation between DNAm and age (Figure 1*B*) indicated that the biological age of donors also is retained in IEOs. Indeed, this was confirmed further by calculating the epigenetic age^23^ of IEOs, which was found to correlate with biological age of the donor even in high-passage IEOs (r_s_ = 0.52) (Figure 1*F*), suggesting epigenetic age is maintained accurately over prolonged in vitro culturing. Interestingly, passage number also was found to be associated significantly with DNAm changes in Principal component (PC)1–3 and 6, indicating an impact of culture duration on epigenetic variation in IEOs (Figure 1*B* and *E*). Plotting PC2 vs PC3 shows that passage number accounts for approximately 10% of variation observed in IEO DNAm profiles (r_s_ = -0.82) (Figure 1*E*).

**Table 1:** Intestinal Organoid Data Sets Used in Analysis

Cohort	Sample number	Data type	Gut segments	Passage	Age	Sex, female %	Source
1	80	DNAm	TI and SC	1–16	Pediatric	55	Newly generated
2	30	DNAm	TI and SC	1–11	Pediatric and adult	50	E-MTAB-4957^19^
3	21	DNAm	Colon, duodenum, jejunum	2–11	Pediatric and adult	48	GSE141256^10^
4	42	DNAm and gene expression	TI and SC	2–12	Pediatric	67	Newly generated

NOTE. Cohorts were either generated for this analysis or are publicly available from the listed sources.

SC, sigmoid colon.

To ensure that observed culture-associated DNAm changes were not a result of laboratory or sample cohort–specific culturing techniques, we tested this phenomenon in additional, publicly available cohorts previously generated by our group (cohort 2), as well as by an independent group (cohort 3) (Table 1). In both additional cohorts, IEOs were generated from mucosal biopsy specimens obtained from the small (TI, duodenum, jejunum) and large bowel (colon) and passage number was recorded. These IEOs represent a wide range of donor ages (Table 1). Genome-wide DNAm was assessed using Illumina arrays (450K and EPIC arrays; Illumina, San Diego, CA; see the Methods section). In total, DNAm was analyzed in an additional 51 mucosa-derived human IEOs. Performing PCA as described earlier on the additional cohorts 2 and 3 confirmed highly significant associations between DNAm changes with passage (Figure 1*G* and *H*).

Together, these results show that although the vast majority of IEO DNAm appears to be stable even during prolonged culture periods, approximately 10% of epigenetic variation is associated strongly with culture duration in both a large discovery as well as 2 additional validation cohorts.

### Passage-Associated Directional DNAm Changes Are Consistent in IEOs Derived From Different Gut Segments and are Validated in Additional IEO Culture Cohorts

**Figure 2:**
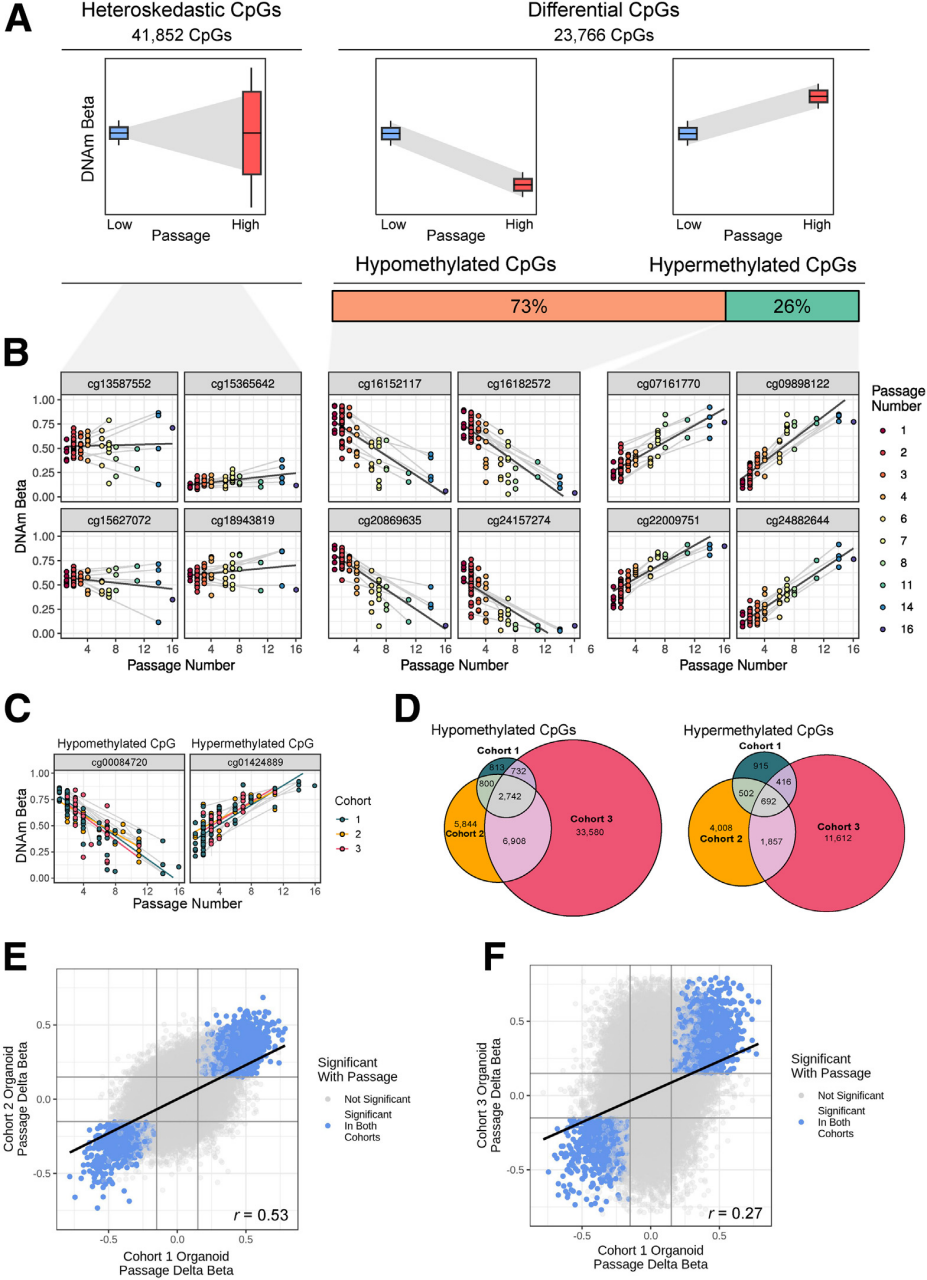
**Long-term culture effects on IEO DNAm are validated in independent cohorts.** (*A*) Schematic of the 3 types of passage-associated DNAm changes at individual CpGs and the proportion of passage CpG split by type of change in DNAm. (*B*) Representative CpGs with DNAm significantly associated with passage. Samples are colored by passage number and *grey lines* connect samples derived from the same patient. Regression lines between passage and DNAm are shown in black. (*C*) Representative CpGs with significant differential DNAm associated with passage in all 3 cohorts. Samples are colored by cohort and *grey lines* connect samples derived from the same patient. Regression lines between passage and DNAm are shown separately for each cohort. (*D*) Overlap of CpGs in cohorts 1–3 with significant differential DNAm associated with passage. (*E* and *F*) Direction of effect is consistent between cohorts. The delta betas from 2 cohorts are shown as points with CpGs significantly associated with passage highlighted.

Having established a reproducible association between DNAm variance with culture duration (ie, passage), we next investigated directional changes of DNAm during prolonged in vitro culture. In general, there are 3 ways by which DNAm can change over time: (1) a stochastic occurrence of either gain or loss of DNAm (ie, heteroskedastic), (2) loss of DNAm (hypomethylation), and (3) gain of DNAm (hypermethylation) (Figure 2*A*). To identify directional DNAm changes, we performed differential DNAm analyses comparing low- (ie, 1–4) with high- (ie, 5–16) passage IEOs (see the Methods section).

Assessing DNAm changes in our discovery cohort 1, we identified 41,852 cytosine-phosphate-guanine dinucleotides (CpGs) (of 798,096 CpGs tested on the EPIC array, 5.2%) that showed significant heteroskedasticity with passage (false discovery rate [FDR], <0.05) (Figure 2*B*). Furthermore, 17,352 CpGs (2.2%) showed loss of DNAm (hypomethylated) while 6414 CpGs (0.8%) gained DNAm (hypermethylated) over time (FDR, <0.05; |delta beta| > 0.15) (Figure 2*B*).

Directional changes in DNAm (ie, gain or loss of DNAm) associated with passage were validated by showing a major overlap of CpGs in both additional cohorts 2 and 3 (FDR, <0.05; |delta beta| > 0.15) (Figure 2*C* and *D*), as well as consistency in the magnitude of passage-associated differential DNAm (Figure 2*E* and *F*).

Taken together, a subset of passage-associated directional DNAm changes were validated in 2 additional cohorts, further suggesting that this phenomenon occurs independently of the sample cohort and laboratory.

### In Vitro Culture Induces Functional and Transcriptional Changes of IEOs That Are Partly Associated With DNAm Changes

**Figure 3:**
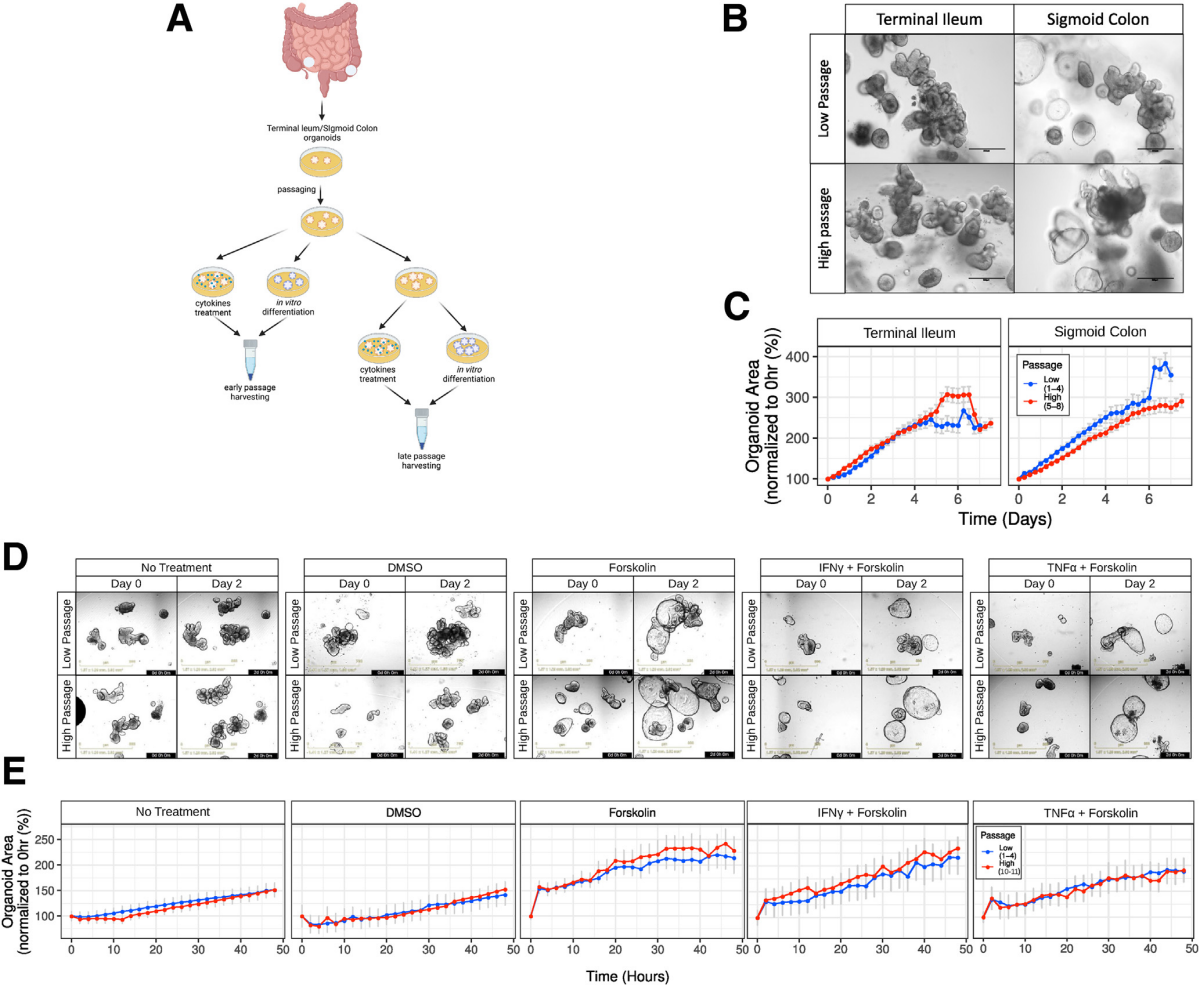
**High- and low-passage IEOs are functionally similar.** (*A*) Outline of experimental design. (*B*) Bright-field images of TI- and sigmoid colon–derived IEOs at low and high passage taken by the EVOS FL system (Life Technologies). *Scale bars*: 300 μm. (*C*) Comparison of growth curves between low- and high-passage TI- and SC-derived IEOs (n = 2, 6 technical replicates per each biological replicate). (*D*) Representative images of TI organoids in standard medium Non-treated (NT), vehicle control medium (DMSO), cultured with forskolin IFNγ+forskolin or TNFα+forskolin taken with by Incucyte. *Scale bars*: 800 μm. (*E*) Comparison of organoid size between early and late-passage organoids after proinflammatory cytokines and forskolin treatments (n = 2, 4 technical replicates per each biological replicate).

Having observed distinct DNAm changes associated with prolonged culturing of IEOs, we next aimed to investigate the potential impact of culture duration on cellular function and gene transcription. We, therefore, generated an additional cohort (cohort 4) of IEOs from small- and large-bowel biopsy specimens from 5 healthy individuals (Figure 3*A*). IEOs were kept in culture for up to 12 passages (approximately 4 months) and subjected to a range of functional assays as well as genome-wide epigenetic (ie, DNAm) and transcriptomic profiling. As shown in Figure 3*B*, culture duration did not impact on the microscopic appearance of IEOs as distinct differences, such as a more budded appearance of small-bowel and cystic appearance of large-bowel/colonic IEOs were retained in high-passage organoids (Figure 3*B*). Similarly, measuring IEO size over several days after passaging showed no difference in growth when comparing IEOs at low vs high passage within each gut segment (*P* < .05) (Figure 3*C*).

Next, we aimed to test whether culture duration impacts barrier function of IEOs at baseline and in response to inflammatory cytokines. We therefore developed a modified forskolin-induced swelling assay, which has been used previously in the context of cystic fibrosis to test the function of cystic fibrosis transmembrane conductance regulator in gut organoids (see the Methods section).^24^^,^^25^ Briefly, the assay takes advantage of the ability of forskolin to increase cyclic adenosine monophosphate levels in the intestinal epithelium. This in turn results in the opening of iron channels followed by transport of ions and water into the lumen of organoids, causing them to swell up. IEOs with an intact barrier will swell up over time while any damage to barrier function will either stop IEOs from swelling and/or limit their capacity of swelling. IEO size therefore correlates with barrier function (Supplementary Video 1). IEOs incubated with either tumor necrosis factor α (TNFα) or interferon γ (IFNγ) and forskolin for 48 hours showed significantly reduced swelling compared with IEOs incubated with forskolin only (*P* < .05), suggesting that these cytokines impact on epithelial barrier function as expected (Figure 3*D* and *E*). Importantly, culture duration did not impact the epithelial cell barrier because we did not observe any difference between high- and low-passage IEOs within each gut segment (*P* > .05) (Figure 3*D* and *E*).

**Figure 4:**
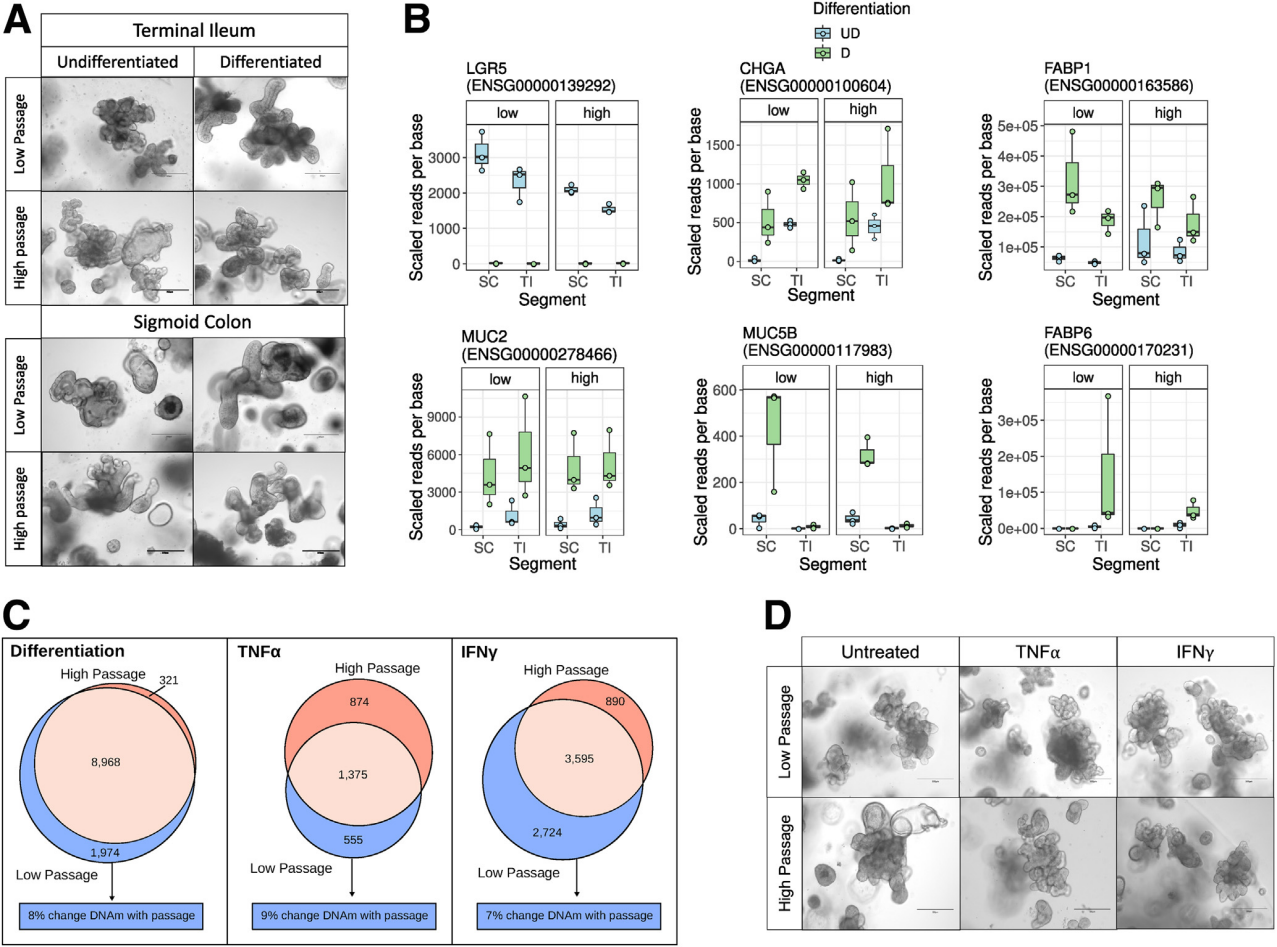
**High- and low-passage IEOs are similar upon differentiation and proinflammatory cytokine stimulation.** (*A*) High- and low-passage IEOs look similar before and after differentiation. Bright-field images of TI- and sigmoid colon (SC)-derived IEOs after in vitro differentiation, respectively, taken by the EVOS FL system (Life Technologies). *Scale bars*: 300 μm. Outline of experimental design. (*B*) Gene expression of selected differentiation markers in IEOs by gut segment and passage (high or low). (*C*) Venn diagram illustrating overlap of differentially expressed genes in high- and low-passage IEOs upon differentiation and co-culture with IFNγ or TNFα. (*D*) Bright-field images of TI-derived IEOs at low and high passage after proinflammatory cytokine treatment (TNFα or IFNγ), taken by the EVOS FL system (Life Technologies). *Scale bars*: 300 μm. D, Differentiated; UD, Undifferentiated.

**Figure 5:**
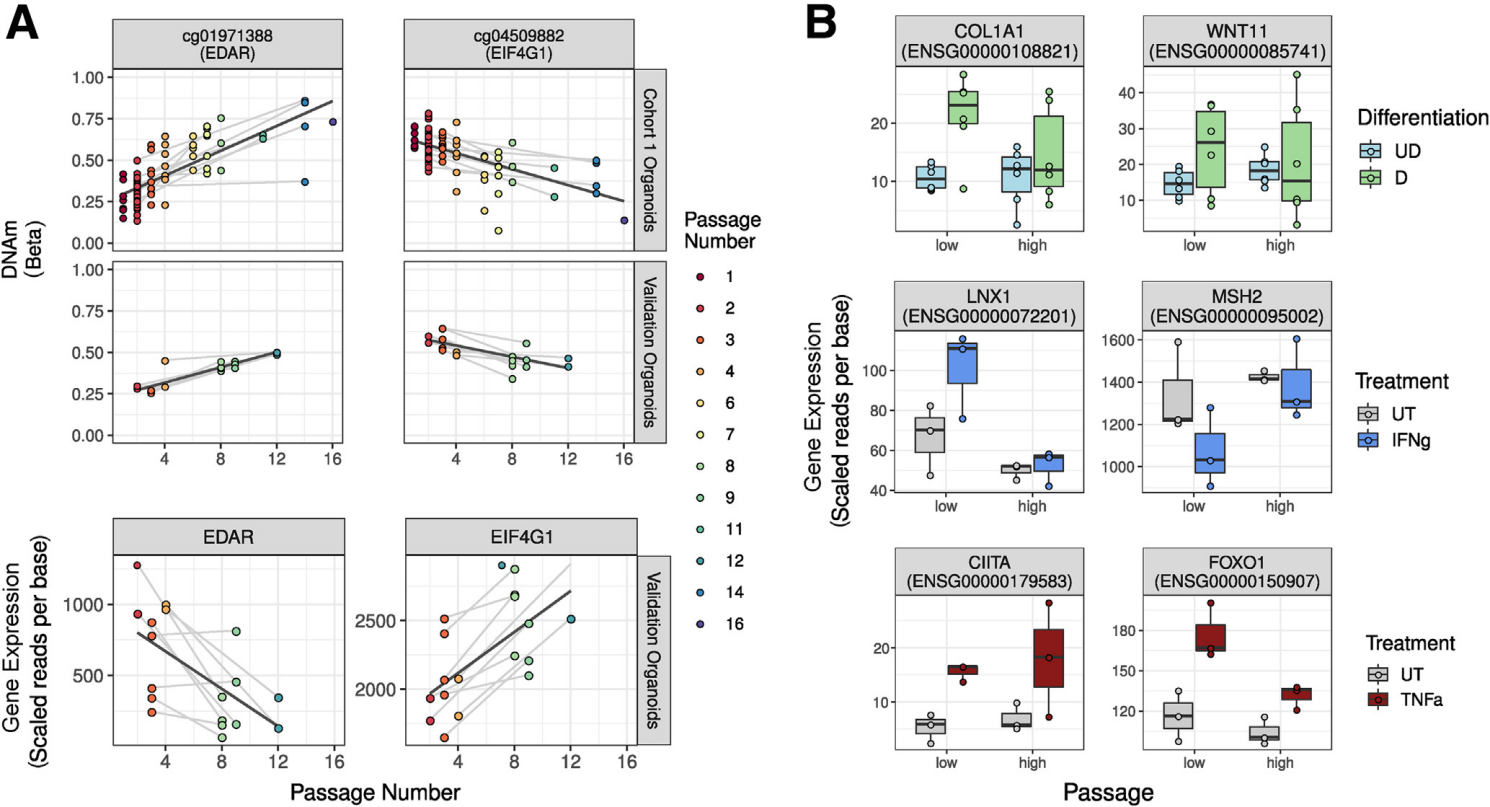
**Genes are differentially expressed with IEO passage.** (*A*) Representative CpGs and genes showing DNAm and mRNA expression associated significantly with passage in cohort 1 and a validation cohort. Samples are colored by passage number and *grey lines* connect samples derived from the same patient. Regression lines between passage and DNAm/expression are in black. (*B*) mRNA expression of representative genes showing passage-dependent differences in response to in vitro differentiation, IFNγ, or TNFα. D, Differentiated; UD, Undifferentiated; UT, Untreated.

To test the potential impact of culture duration on the ability of the human intestinal epithelium to differentiate into cell subsets, IEOs were subjected to in vitro differentiation by withdrawing Wnt agonists over 4 days.^19^ Importantly, passage number did not impact the expression levels of selected gut segment–specific marker genes, or on the microscopic appearance of IEOs (Figure 4*A*). Gene transcription was assessed on extracted RNA using RNA sequencing. As described previously, withdrawal of Wnt agonists led to decreased expression of intestinal epithelial stem cell marker *LGR5* while expression of epithelial cell subset (*MUC5B*) and differentiation markers (*FABP1* and *FABP6*) increased in a gut segment–specific manner (Figure 4*B*). Furthermore, of 10,942 genes found to change expression in response to Wnt agonist withdrawal in low-passage IEOs, expression of 8968 also changed in high-passage IEOs (FDR, <0.05). In total, 8% of genes that were found to change their expression in response to Wnt agonist withdrawal in low- but not high-passage IEOs are associated with at least 1 CpG showing passage-associated DNAm changes (Figure 4*C*, left panel; Supplementary Table 1). To evaluate the potential impact of culture duration on the responsiveness of IEOs to inflammatory stimuli, low- and high-passage IEOs were co-cultured with IFNγ or TNFα for 24 hours, and gene expression was assessed on extracted RNA (Figure 3*A*). As shown in Figure 4*C*, a major overlap was found between differentially expressed genes in response to IFNγ and TNFα comparing low- with high-passage IEOs and no microscopic changes were observed (Figure 4*D*). Similar to transcriptional changes in response to in vitro differentiation, of the genes only changed in low-passage IEOs, 7% and 9% (IFNγ and TNFα, respectively), were associated with at least 1 CpG showing passage-associated DNAm changes, indicating a limited impact of culture-associated DNAm changes on inflammation-induced gene transcription. However, a highly significant association between passage-associated DNAm and transcriptional changes was observed for several genes including *EDAR* and *EIF4G1* (Figure 5*A*, Supplementary Table 2). In addition, some of the genes in which DNAm was associated with passage showed striking differences in gene expression in response to differentiation and stimulation (Figure 5*B*, Supplementary Tables 3 and 4). Interestingly, transcriptional responses to both in vitro differentiation and exposure to IFNγ were larger in early vs late-passage IEOs, suggesting that culture duration may impact the overall magnitude of response in the intestinal epithelium.

In summary, although the impact of culture duration on microscopic appearance and gross cellular function of IEOs appears to be limited, distinct transcriptional changes were observed and some of them are associated with DNAm.

### Culture-Associated DNAm Changes in IEOs Exclude Hypomethylated Promoter Regions and Share Features of Intestinal Cancer

Having determined the impact of passage-associated DNAm changes on gene transcription and cellular function of IEOs, we next aimed to investigate their distribution patterns across the genome.

**Figure 6:**
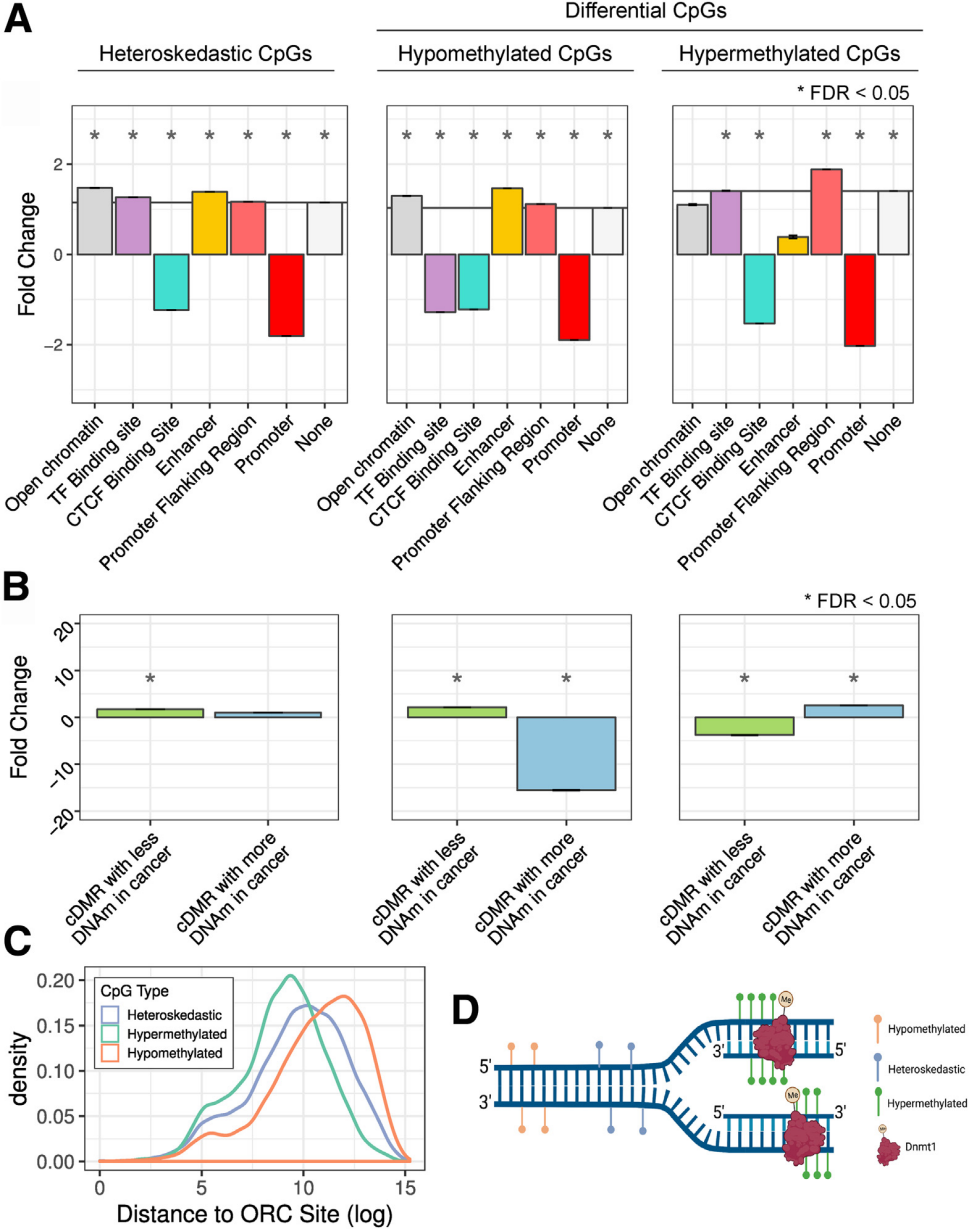
**Passage affects DNAm in specific regions of the genome.** (*A* and *B*) Enrichment of passage-associated CpGs across genomic regions expressed as fold change relative to the total number of CpGs present on the EPIC array. Standard error bars indicate mean fold change for the error across 1000 random samplings. (*C*) Distance of passage-associated CpGs from origin replication complex (ORC) sites. (*D*) Schematic showing location of passage-associated DNAm changes relative to the origins of replication.

As shown in Figure 6*A*, heteroskedastic CpGs were enriched in transcription factor (TF) binding sites, open chromatin, enhancers, and promoter flanking regions, but depleted in promoters and CCCTC-binding factor (CTCF) binding sites^26^ (FDR, <0.05). Furthermore, CpGs losing DNAm were enriched significantly in open chromatin, enhancers, and promoter flanking regions, but depleted in promoters, CTCF binding sites, and TF binding sites (FDR, <0.05) (Figure 6*A*). In contrast, CpGs gaining DNAm in culture were enriched in TF binding sites and promoter flanking regions, but also depleted in promoters and CTCF binding sites (FDR, <0.05) (Figure 6*A*), suggesting that the dysregulation of DNAm with passage occurred genome wide, but seemed to spare nonvariable unmethylated regions.

Pathways possibly affected by passage-associated DNAm changes were explored through enrichment of any gene ontology (GO) gene sets in the genes adjacent to passage-associated DNAm changes. Hypomethylated and heteroskedastic CpGs were enriched in similar GO gene sets, including adherens junction organization (GO:0034332) and cell–cell adhesion via plasma-membrane adhesion molecules (GO:0098742) (Supplementary Tables 5 and 6), and these gene sets had little overlap in the hypermethylated gene sets (Supplementary Table 7). In the heteroskedastic and hypomethylated overlapping gene sets there were also many unexpected GO sets, such as neuron projection guidance (GO:0097485) and synapse assembly (GO:0007416). These gene sets could be enriched because the genome is hypomethylated globally and the genes enriched are not specific to epithelial functional pathways. Alternatively, there may be unknown functions for these genes in gut epithelial cells.

Global loss and local gain of DNAm have been linked to various malignancies including colon, breast, and prostate cancers.^27^, ^28^, ^29^, ^30^ We next looked at a previously characterized list of differentially methylated regions seen in colon cancer (cDMRs).^20^ Interestingly, we found that within these cDMRs, passage-associated DNAm changes in IEOs followed the same pattern (ie, gain or loss of DNAm) as those reported in colon cancer (Figure 6*B*). Moreover, CpGs that lost or gained DNAm in high-passage IEOs were enriched in cDMRs that lost or gained DNAm in cancer, respectively (FDR, <0.05) (Figure 6*B*). Heteroskedastic CpGs also were enriched in cDMRs hypomethylated in cancer (FDR, <0.05) (Figure 6*B*).

It has been proposed in the context of cancer that late-replicating regions of the genome will have less time to remethylate CpGs on the daughter strand after DNA replication (Figure 6*C*).^31^ A replication-associated loss of DNAm also was observed in cell culture models.^32^ We, therefore, tested if observed passage-associated DNAm changes could be caused by inferred DNA replication timing. Using 52,251 origins of replication locations based on origin replication complex 2 binding sites,^33^ we found that CpGs that lose DNAm with passage were located further from origins of replication than expected by chance (*P* < .001) (Figure 6*D*). Interestingly, the same was true for heteroskedastic CpGs, while hypermethylated CpGs were located closer to origins of replication than expected by chance (*P* < .001) (Figure 6*D*). Taken together, these results suggest that passage-associated DNAm changes in human IEOs share features of epigenetic changes observed in colon cancer and may be caused at least in part by fast cell turnover in vitro.

## Discussion

Since the establishment of human mucosa–derived IEO as powerful translational research tools just over a decade ago, the number of applications has continued to increase dramatically. Major new areas of interest include precision and regenerative medicine, drug discovery and development, as well as modeling of disease pathogenesis.^1^, ^2^, ^3^, ^4^, ^5^ Importantly, the vast majority of applications require IEOs to be cultured for prolonged time periods to sufficiently expand cell numbers for larger scale experiments. Moreover, the ability to culture IEOs over longer time periods (ie, months) is also essential for the testing of specific chronic stimuli (eg, inflammation or infection) on epithelial cell function over time. Although numerous studies have confirmed the ability to maintain human mucosa–derived IEOs in culture over prolonged time periods, there still is little information available on the potential impact of culture duration on cellular function.

Epigenetic mechanisms can alter cellular function in response to environmental changes. As one of the main epigenetic mechanisms in mammalian cells, DNAm is known to play a key role in regulating intestinal epithelial cellular function. In this study, we show distinct culture-associated DNAm changes in human mucosa–derived IEOs occurring over several months regardless of from which gut segment or human donor the IEOs were derived. Although the vast majority of CpGs tested was found to be highly stable, including those specific for age and anatomic location, approximately 8% of probes showed DNAm changes that were associated with culture. Although a proportion of methylation changes could be caused by limitations relating to the methodologies used, we were able to validate a large proportion of culture-associated DNAm changes across several IEO cohorts that were generated by independent groups. Our findings are in keeping with previous reports on directed and stochastic changes to DNAm with passaging of induced pluripotent stem cells.^34^ Specifically, induced pluripotent stem cells were reported to lose DNAm with increased passaging; tissue-specific DNAm patterns observed in early passages were lost, and cells converged to an embryonic stem cell–like state of DNAm.^34^ The effect of passage on DNAm also has been examined in the context of cellular senescence, in which methylation patterns in high-passage cells were compared with immortalized cell lines.^35^^,^^36^ In immortalized lines, DNAm undergoes stochastic changes after immortalization, whereas cells passaged to senescence underwent a programmed set of changes in DNAm consistent across samples.^35^^,^^36^ Considering published evidence, our findings suggest that culture-associated DNAm changes also may occur to varying degrees in other pluripotent or adult stem cell–derived human organoid culture models.^6^^,^^37^

A key question arising from our findings relates to the likely cause of DNAm changes observed in IEOs. Epigenetic changes occurring over time, or with age, generally are referred to as epigenetic drift. According to our current understanding, the main mechanisms involved in causing epigenetic drift include genetic variation, environmental factors, as well as stochastic changes as a result of inaccurate copying of methylation signatures during cellular divisions.^38^, ^39^, ^40^ Given previously reported genetic stability of IEOs in culture,^41^^,^^42^ environmental changes and random variation are most likely the main drivers. Indeed, given major differences in the microenvironment between the intestinal stem cell niche in vivo and in vitro, the occurrence of epigenetic changes is not surprising. Furthermore, culture conditions used to rapidly expand IEOs may increase cell turnover, thereby introducing DNAm changes because of stochastic errors in copying DNAm signatures from intestinal stem cells to daughter cells. Although this issue could be addressed, for example, by reducing the concentration of Wnt agonists in the culture medium, such changes would result in increased time periods required to expand organoids sufficiently for downstream analyses. Other modifications to culture conditions such as supplementation with methyl donors (eg, folate) also may help to slow down or avoid DNAm changes.

Arguably the most important question we aimed to answer in our study is whether observed DNAm changes alter gene transcription or cellular function of IEOs. Reassuringly, we found that the overall impact of culture-associated DNAm changes in human IEOs was limited and did not impact cellular identity (eg, gut segment–specific methylation signatures), epigenetic age, or broad cellular function such as growth and intestinal barrier function. However, performing simultaneous genome-wide transcriptional and epigenetic profiling of IEOs showed a subset of passage-associated DNAm changes that correlated with changes in gene expression at baseline, as well as in response to in vitro differentiation and exposure to inflammatory cytokines. The latter is particularly relevant and important for the studies using organoids as models to investigate related inflammatory conditions such as inflammatory bowel disease. Interestingly, we also identified highly significant gene expression changes at baseline and in response to treatment with inflammatory cytokines, which were not associated with DNAm changes. These changes could be caused by other epigenetic mechanisms such as post-translational histone modifications or expression of small noncoding RNAs and future studies are required to investigate this important question.

Another key finding in our study was the observed global loss of DNAm in IEOs associated with prolonged in vitro culture, which is a recognized feature of various malignancies including colorectal cancer. Indeed, we identified several additional similarities including the overlap and directional methylation change between passage-associated DMRs with known cDMRs, as well as the significant proportion of heteroskedastic methylation changes. The latter has been linked to higher variability in DNAm of colon cancer compared with normal samples.^43^ Furthermore, global loss of DNA methylation in cancer has been attributed to incomplete remethylation of CpGs during mitosis, possibly as a result of the higher cell turnover.^31^ In keeping with this hypothesis, CpGs that lose DNAm as part of malignant transformation frequently are found in late replicating regions of the genome. This lends further support to the hypothesis mentioned earlier that rapid cellular turnover of human IEOs in vitro may contribute in part to observed epigenetic changes, some of which share similarities to malignant cellular transformation.

We recognize the limitations of our study, including those associated with methodologies used to perform genome-wide DNAm analyses of IEOs. Although Illumina methylation arrays (ie, 450K and EPIC) are established and validated tools to comprehensively evaluate promoter regions, CpG islands, as well as other potentially relevant intergenic regions, they only cover 3% of the approximately 28 million CpG sites present in the entire human genome. Furthermore, because culture duration between passaging of IEOs varies we are unable to provide exact recommendations on when DNAm changes occur. However, it is clear from our results that the risk for epigenetic changes to develop is linked directly to culture duration. Indeed, our study includes IEOs that were passaged up to 16 times, relating to approximately 5 months in culture. Because organoids can be and frequently are cultured for substantially longer time periods (eg, >1 year), we would expect to see epigenetic and/or associated functional changes accumulating over time, further emphasizing the need to consider culture duration as a critical part of experimental design.

In summary, our study identified distinct, culture-associated DNA methylation changes in human mucosa–derived IEOs that impact gene transcription and cellular function and share features of malignant transformation. Although global epithelial cell function was found to be retained, our findings highlight the critical importance of considering culture duration in the experimental design and interpretation of data derived from human IEOs.

## Methods

### Patient Recruitment and Sample Collection

Intestinal biopsy specimens were collected from the TI and sigmoid colon from 52 children aged 1 to 16 years undergoing diagnostic endoscopy. This study was conducted with informed patient and/or carer consent as appropriate, and with full ethical approval (REC-12/EE/0482).

### Human IEO Culture

Human IEOs were generated from mucosal biopsy specimens by isolation of intestinal crypts and culturing in Matrigel (Corning, NY) using media described previously.^7^^,^^19^^,^^37^^,^^44^ The medium was replaced every 48–72 hours and once the IEOs were well established, they were passaged every 7–10 days by mechanical disruption and reseeded in fresh Matrigel. IEOs cultured up to passage number 4 were considered low-passaged organoids, while IEOs cultured from passage number 5 to 16 were considered high-passaged organoids. This threshold was chosen for a number of reasons. First, we routinely require a minimum of 4 passages to expand organoids sufficiently for most downstream analyses including the generation of frozen stocks. Second, as part of extensive data analyses we performed mixture model thresholding that indicated major differences in the DNAm profiles in IEOs cultured for more than 4 passages.

### In Vitro Differentiation and Co-culture With Proinflammatory Cytokines of Human IEOs

In vitro differentiation of human IEOs was performed by culturing IEOs in standard growth medium for 4 days followed by removal of Wnt agonists (referred to as *differentiation medium*) (Table 2) for an additional 4 days. For the treatment of human IEOs with proinflammatory cytokines, IEOs were cultured for 5 days after splitting in the growth medium, followed by 24 hours of treatment with recombinant human protein TNFα (H8916; Sigma Aldrich, Burlington, MA) at 40 ng/mL or IFNγ (PHC4031; Life Technologies, Carlsbad, CA) at 20 ng/mL. Bright-field images were taken using an EVOS FL system (Life Technologies).

**Table 2:** Differentiation Medium Components

Differentiation medium (total volume, 12.5 mL)	Final concentration
Advanced DMEM/F12+++	97.44% (vol/vol)
Primocin (Invivogen San Diego, CA)	500 μg/mL
B-27 R supplement (Invitrogen, Carlsbad, CA)	1×
N-acetylcysteine (Sigma, St. Louis, MO)	1.25 mmol/L
A3801 (Tocris, Bristol, UK)	500 nmol/L
SB202190 (Sigma)	10 μmol/L
Murine EGF (Invitrogen)	50 ng/mL
Murine Noggin (Peprotech, Rocky Hill, NJ)	100 ng/mL

EGF, Epidermal Growth Factor; DMEM, Dulbecco’s modified Eagle medium.

### Human IEO Growth and Barrier Integrity Assessment

Human IEO growth was assessed using the Incucyte SX5 (Sartorius AG, Göttingen, Germany) by imaging every 6 hours over 7 days for each passage. After 7 days, the images were analyzed using the Incucyte IEO analysis software, which allowed measurement of the IEO area over time. For each human IEO line and for each passage, 6 wells were imaged and analyzed to generate an average measurement of IEO growth. Comparisons between IEO passages were made using analysis of variance.

Human IEO barrier integrity was evaluated by culturing the IEOs at early and late passages, as described earlier, from day 0 to day 4. On day 5, IEOs were collected from 48-well plates and transferred to 96-well plates, seeding 5–10 IEOs per well in 5 μL Matrigel and 100 μL growth medium. Using 3 wells per condition, human IEOs were cultured or in standard condition medium, or in vehicle control medium (+dimethyl sulfoxide), or in forskolin (5 μmol/L) medium in the presence or absence of IFNγ (20 ng/mL) or TNFα (40 ng/mL). The plates were placed in the Incucyte SX5 to be imaged every 2 hours for 48 hours. After 2 days, the experiment was stopped and images were analyzed to measure IEO area over time using the Incucyte IEO analysis software.

### Harvesting of Human IEOs and DNA and RNA Extraction

At the end of each experiment, human IEOs were harvested and both DNA and RNA were extracted using the AllPrep DNA/RNA mini kit (Qiagen, Hilden, Germany). DNA was bisulfite-converted using the EZ DNA methylation Gold kit (Zymo Research, Irvine, CA).

### DNAm Profiling and RNA Sequencing

Genome-wide DNAm was profiled using the Illumina EPIC platform (Illumina), and deposited in ArrayExpress (EMBL-EBI, Hinxton, UK) under accession numbers E-MTAB-9748 and E-MTAB-11545. An overview of sample numbers can be found in Table 1.

Expression profiling was performed using RNA sequencing by Cambridge Genomic Services (University of Cambridge) and can be found in ArrayExpress under accession number E-MTAB-11548. The code for analysis is available at: redgar598.github.io/DNAm_organoid_passage.

### Access to Data

All authors had access to the study data and reviewed and approved the final manuscript.

### DNAm Data Preprocessing and Quality Control

DNAm data were processed using the minfi package,^45^ specifically the preprocess function to extract β values from IDAT files. Data then were normalized based on control probes on each array using functional normalization.^46^ Removal of 2 samples as outliers and those failing basic sense checks resulted in 80 IEO samples derived from 46 individuals. Starting with the 866,238 probes on EPIC, probes were filtered if they assayed a polymorphic CpG,^47^ were on a sex chromosome, had a demonstrated potential to cross-hybridize to several regions of the genome,^47^ or had a detection *P* value greater than .05 in 1% of samples. This filtering left 798,096 CpGs for analysis.

### Correlation of IEO DNAm With Passage

The association between DNAm and culture duration (quantified by passage) was investigated with PCA. The loadings of each PC were associated with technical and biological variables using analysis of variance for categoric variables or Spearman correlations for continuous variables. An association between DNAm and passage also was tested on an individual CpG level. Differential DNAm with increasing passage number was tested using linear models at each of 798,096 CpGs and significant heteroskedasticity in DNAm was tested with a Breusch–Pagan test.

### Public DNAm Data

Publicly available data sets^10^^,^^19^^,^^48^ used in this study are summarized in Table 1. The data were processed in the same way as described for cohort 1, except using a different array annotation for data from the 450K array instead of EPIC.^49^ In cohort 3 both 450K and EPIC arrays were used so only the 384,188 CpGs on both arrays, after probe filtering, were used. Batch correction for array type was performed using ComBat.^50^

### Matched DNAm and Gene Expression Cohort

The DNAm data were measured and processed as for cohort 1 (Table 1). In the 18 untreated undifferentiated IEOs, differential DNAm with increasing passage number was tested with a linear model with a covariate for donor. For differential DNAm with differentiation and proinflammatory cytokine treatments, samples were split into low or high passage, and then within those groups a linear model with a covariate for donor was used to identify any differential CpGs.

### Enrichment DNAm Passage Changes in Genomic Features

Enrichment of CpGs that showed DNAm changes with passage in various genomic features was tested. Analyses were performed separately for CpGs showing hypermethylation and hypomethylation with increased passage as well as a heteroskedastic DNAm pattern. The differential and heteroskedastic CpGs identified were explored for enrichment in genomic regulatory features. The Ensembl Regulatory Build^26^ was collected for GRCh37 using BioMart^51^ (retrieved November 2019). CpGs on the EPIC array were annotated as overlapping any of the 6 regulatory regions or as not in any annotated regulatory region. Enrichment *P* values for differential and heteroskedastic CpGs, in each regulatory region, were calculated using 1000 randomly sampled lists of CpGs, to account for the underlying distribution of CpGs on the EPIC array. For hypomethylated and hypermethylated CpGs a change in DNAm of 0.15 of -0.15 was required. Therefore, the background of CpGs was modified to exclude those with a DNAm value >0.15 for hypomethylated CpGs and <0.85 for hypermethylated CpGs in the passage 1 IEO. These 223,695 and 295,469 CpGs, respectively, could never pass the threshold of change in DNAm and should not be included in the background CpGs list.

Similarly, enrichment *P* values were generated for CpGs in previously described cDMRs.^20^ Then, to assess distance from origins of replication, the absolute minimum distance of a CpG from a boundary of an origin of replication (origin replication complex 2) binding peak^33^ was used. Finally, the mean distance of CpGs associated with passage was compared with the means of 1000 randomly sampled lists of CpGs on the EPIC array, as described earlier for regulatory region associations.

### RNA Sequencing Data Analyses

For each of the 42 validation samples, RNA was prepared with the Truseq mRNA library preparation (Illumina, San Diego, CA) and sequencing was performed on NextSeq (Illumina, San Diego, CA) 75-cycle high output. RNA sequencing data were quality controlled using FastQC.^52^ Reads were pseudoaligned using kallisto^53^ indexed human transcriptome (GRch38) and quantified with 100 bootstraps. Using sleuth,^54^ differential expression was measured at the gene level by aggregating across all transcripts associated with a gene (Ensembl Genes 104).^55^^,^^56^ Gene expression was associated with passage as a continuous measure (2–12 passages) using a likelihood-ratio test with a covariate for donor. For differential expression with differentiation and proinflammatory cytokine treatments, samples were split into low or high passage, then within those groups a likelihood-ratio test with a covariate for donor was used with an FDR < 0.05 considered significant.

### Pathways Affected by DNAm Passage Changes

To explore possible pathways affected systematically by passage, the CpGs differentially DNAm with passage were associated with genes, and these genes then were tested for enrichment in GO gene sets. A CpG was assigned to a gene based on proximity to a transcript from Ensembl Genes 99 GRCh37.p13 collected from BioMart.^51^ Enrichment of GO terms in the list of passage-associated genes (heteoskedastic, 13,267 genes; hypomethylated, 5579 genes; hypermethylated, 3673 genes) were tested using overrepresentation analysis in ErmineJ.^57^ Significance of a GO term is reported as the FDR, computed using the Benjamini–Hochberg method in ErmineJ. Also included are the multifunctionality scores of GO terms.^58^
