# Supplementary figures and images for: Culture-Associated DNA Methylation Changes Impact on Cellular Function of Human Intestinal Organoids
Source: Cell Mol Gastroenterol Hepatol. 2022 Aug 28;14(6):1295–310. doi: 10.1016/j.jcmgh.2022.08.008 (PMC9703134; doi:10.1016/j.jcmgh.2022.08.008)

## Slide 1
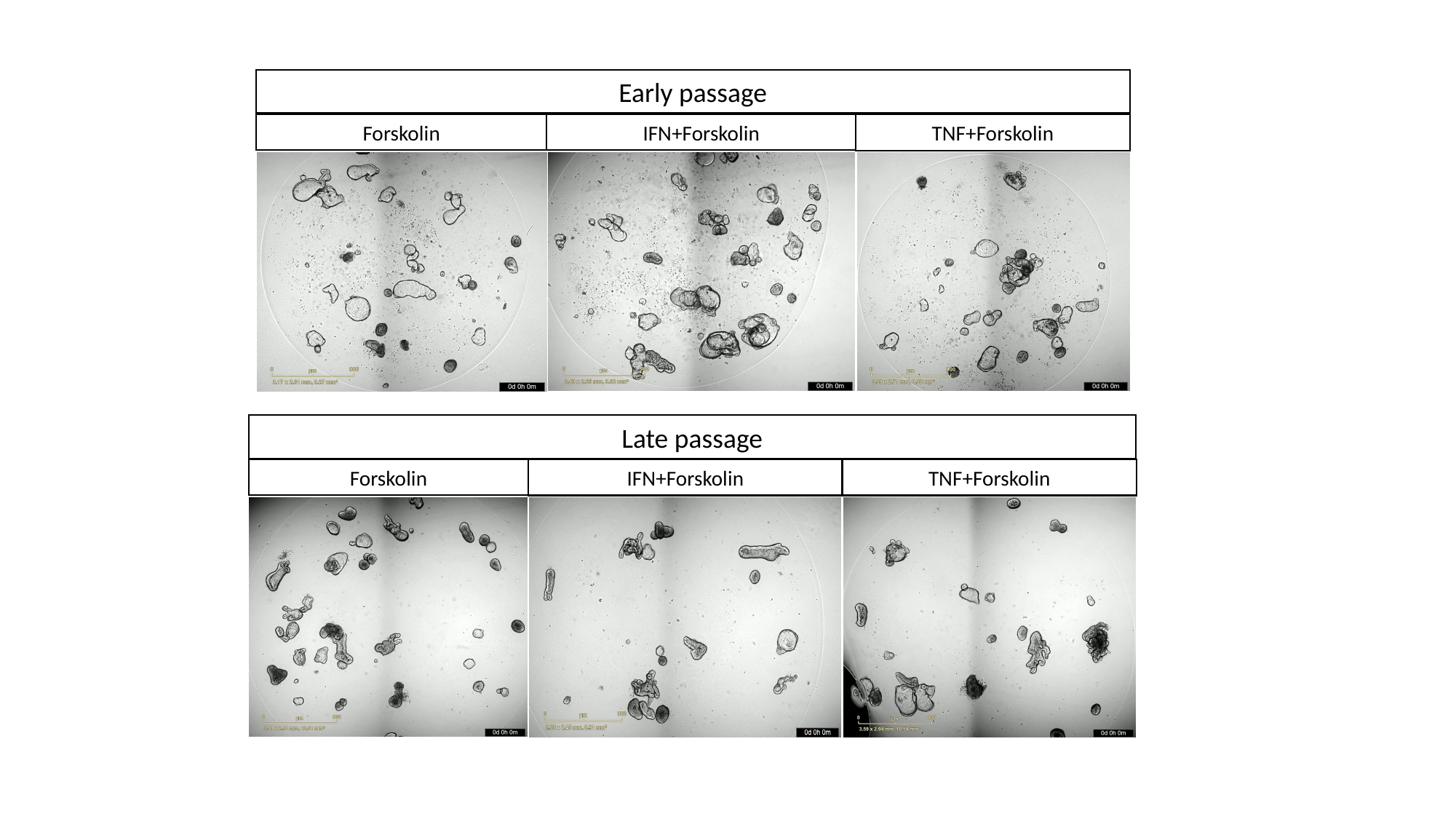

Early passage
Forskolin
Late passage
Forskolin

Supplement: Supplementary Video 1 [file mmc1.pptx]
